# Correlation between seismic activity and tidal stress perturbations highlights growing instability within the brittle crust

**DOI:** 10.1038/s41598-022-11328-z

**Published:** 2022-05-02

**Authors:** Davide Zaccagnino, Luciano Telesca, Carlo Doglioni

**Affiliations:** 1 Sapienza University, Earth Sciences Department, Rome, 00185 Italy; 2 Institute of Methodologies for Environmental Analysis, National Research Council (CNR-IMAA), Tito Scalo (PZ), 85050 Italy; 3 Istituto Nazionale di Geofisica e Vulcanologia (INGV), Rome, 00143 Italy

**Keywords:** Natural hazards, Solid Earth sciences

## Abstract

Faults become more and more responsive to stress perturbations as instability mounts. We utilize this property in order to identify the different phases of the seismic cycle. Our analysis provides new insights about the features of impending mainshocks, which are proposed to emerge from a large-scale crustal-weakening preparation process whose duration depends on their seismic moments, according to the power-law T $\propto$ M$_{0}^{1/3}$ for M$_{0}$ $\le$ 10$^{19}$ N m. Moreover, further studies are performed about the impact of tidal stress perturbation on seismicity; in particular, the relationship between frequency-magnitude scaling and perturbations is discussed, showing that the sensitivity of earthquakes to solid Earth tides decreases as their magnitudes increase.

## Introduction

Several research works prove a significant responsiveness of seismicity to additional stress sources (e.g.,^1^). A nonlinear dependence of the time to failure on stress variations has been known since the Eighties^2^. It does not only mean that seismic rate is a direct effect of loading, but also implies that small additional stress can result in highly unpredictable states of crustal instability. Observations suggest that seismicity rates can be influenced by both static and dynamic perturbations, although in different ways^3^. Tides are ubiquitous periodical stress perturbation sources featured by harmonics with a wide spectrum of periods ranging from a few hours to decades. This is the reason why tidal stress loading can be a key for highlighting different stages of the seismic cycle, i.e., interseismic, pre-seismic, post-seismic phases. Unfortunately, from a statistical viewpoint, earthquake catalogs are often insufficient to detect significant modulations of seismic activity over time with respect to stress modulations. Tides are tiny perturbations of the gravitational field (usually $\sim$ 0.1−10 kPa) with respect to typical earthquake stress drops (1−50 MPa), so that usually a few thousand events are required to observe a statistically significant correlation between tidal phase and earthquakes occurrence. However, the actual impact on the stability of rock volumes largely depends on the tectonic setting, the spatial orientation of the fault, the depth and the epicentral latitude; finally, also the magnitude of the impending event modifies the response of the system to the tidal perturbation (compare with our results in section “Discussion”). At last, seismic response to tidal loading is strongly affected by the duration of earthquake nucleation^4^. Therefore, it is not surprising that a wide range of results was found in different geographical areas. Beyond the aforementioned issues, well-established scientific evidence exists about tidal synchronization in seismic catalogs^5^ as well as the correlation of seismicity with solid Earth tides has been now well documented (e.g.,^6,7^). Both global and regional seismic time series show tidal^8^, climatic and seasonal patterns^9^. The effect of tidal stress can clearly be distinguished into its vertical and horizontal components; in particular, the last has been suggested to provide the energetic tectonic source that is retained by the crustal volume as an hysteresis of the tidal wave passage^10^, whereas the vertical component appears mainly as a transient oscillation of the gravitational load, which acts as the seismic trigger when the threshold of the critical stress is reached also acting in different ways according to the tectonic settings. It has been shown that thrust-related earthquakes are more frequent during the high tide^11^, whereas normal fault related earthquakes occur more often during the low tide^12^. This is mechanically coherent respectively with a decrease of *g* and consequently of the lithostatic load given by *dgz*, where *d* is the crustal density, *g* is the gravity of Earth and *z* represents the depth of the hypocenter, in contractional tectonic settings ($\sigma _{3}$), favouring fault activation, and opposingly by an increase of *g* and the lithostatic load ($\sigma _{1}$) in extensional tectonic settings^13^. In this work, we perform an analysis of the correlation time series of seismic activity and tidal stress perturbations resolved on the fault plane for several areas paying attention to recent seismic activity in Southern California, occurring along strike-slip faults, New Zealand, mainly featured by a transpressional tectonic setting, and Central Italy, mostly characterized by normal faulting. Solid Earth tides turn out to play a dominant role.

## Results

### Southern California

**Figure 1:**
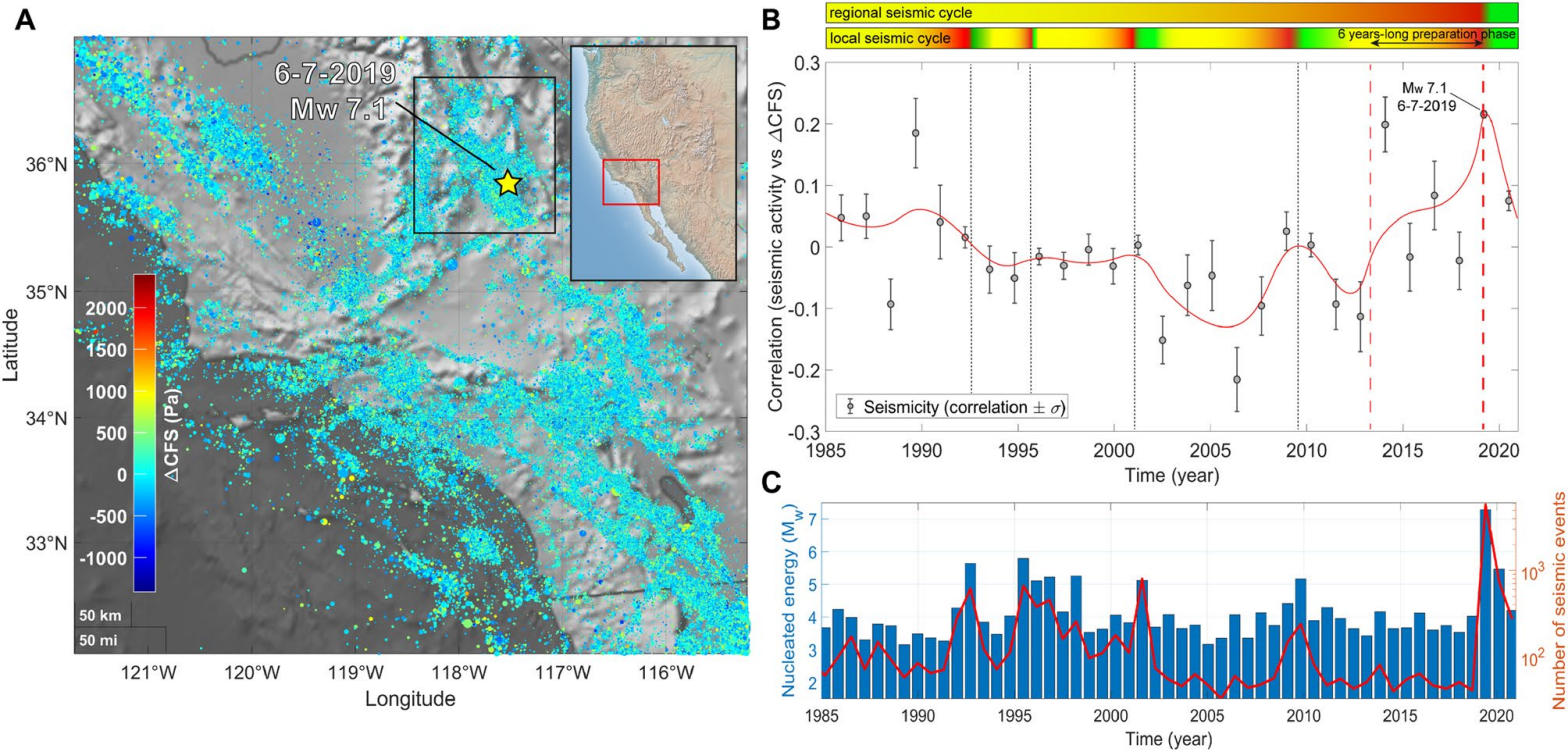
(**A**) $\Delta$CFS map for seismicity in Southern California, SCEDC Catalog, 1985−2021, M$_{L} \ge$ 1.0. Seismicity occurs, on average, at slightly positive $\Delta CFS$ values. The map is realized using the functions “geoscatter” and “geobasemap” in the Matlab environment^63^. (**B**) Correlation $\rho$ between $\Delta$CFS and seismicity in the Ridgecrest District, California, between 1985 and 2021. The scatter plot is realized by taking into account earthquakes happened at latitude 35.2°−36.4° N, longitude 117.0°−118.2° W and -2−20 km deep, M$_{L}$ $\ge$ 1.0. A preseismic phase featured by an increase of the correlation is detected before the Ridgecrest seismic sequence, mainshock 6/7/2019, M$_{w}$ 7.1. The red line is a weighted smoothing spline. Two horizontal color bars above the scatter-plot show nested “seismic cycles” whose phases are highlighted by different colors (green for the post-seismic phase, the interseismic one is in yellow and the coseismic phase is in red). (**C**) Histogram of energy nucleated by earthquakes with M$_{L} \ge$ 1.0 (blue bars) and plot of the number of recorded seismic events (red line).

Our analysis focuses on the region of Ridgecrest, where destructive seismic activity occurred in 2019. On 4$^{th}$ July Southern California was shaken by a M$_{w}$ 6.4 earthquake. It had been preceded by a brief swarm of small magnitude (M$_{w}$ $\le$ 4.1) foreshocks. The next day, an even larger M$_{w}$ 7.1 shallow strike-slip event happened on an orthogonal fault^14^. Correlation time series we realized, compare with Fig. 1, shows a progressively raising correlation, $\rho$, between the tidal Coulomb failure stress, $\Delta$CFS, and energy nucleation that began in between 2012 and 2013 and reached its peak in 2019, $\rho \sim$ 0.24; after that, a sudden drop is observed. In the same region, the Ridgecrest seismic sequence was also forerun by an about ten-years-long decrease of the *b*-value, denouncing mounting instability stress, above all in the area of the M$_{w}$ 6.4 earthquake^15^.

### New Zealand

**Figure 2:**
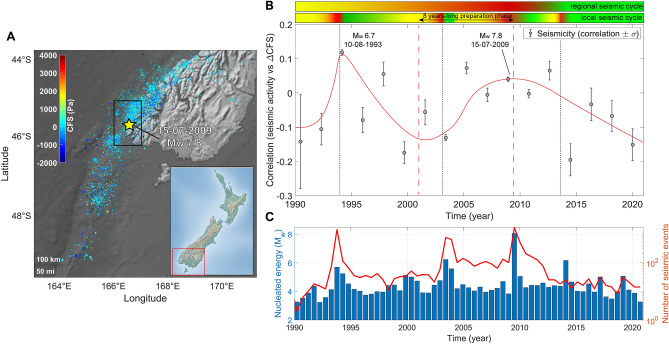
(**A**) $\Delta$CFS map for seismicity in Fiordland region, South Island, New Zealand, GeoNet Catalog, 1990−2021, M$_{L} \ge$ 2.5. The map is realized using the functions “geoscatter” and “geobasemap” in the Matlab environment^63^. (**B**) Correlation $\rho$ between $\Delta$CFS and seismicity in Fiordland, New Zealand (latitude 45.3°−46.2° S and longitude 166.0°−167.0° E, depth 0−33 km) between 1990 and 2021. An about six years long preseismic phase is detected before the 2009 Fiordland earthquake. The red line is a weighted smoothing spline. Two horizontal color bars above the scatter plot show nested “seismic cycles” whose phases are highlighted by different colors (green for the post-seismic phase, the interseismic one is in yellow and the coseismic phase is in red). (**C**) Histogram of energy nucleated by earthquakes with M$_{L}$ $\ge$ 2.5 (blue bars) and plot of the number of recorded seismic events (red line).

**Figure 3:**
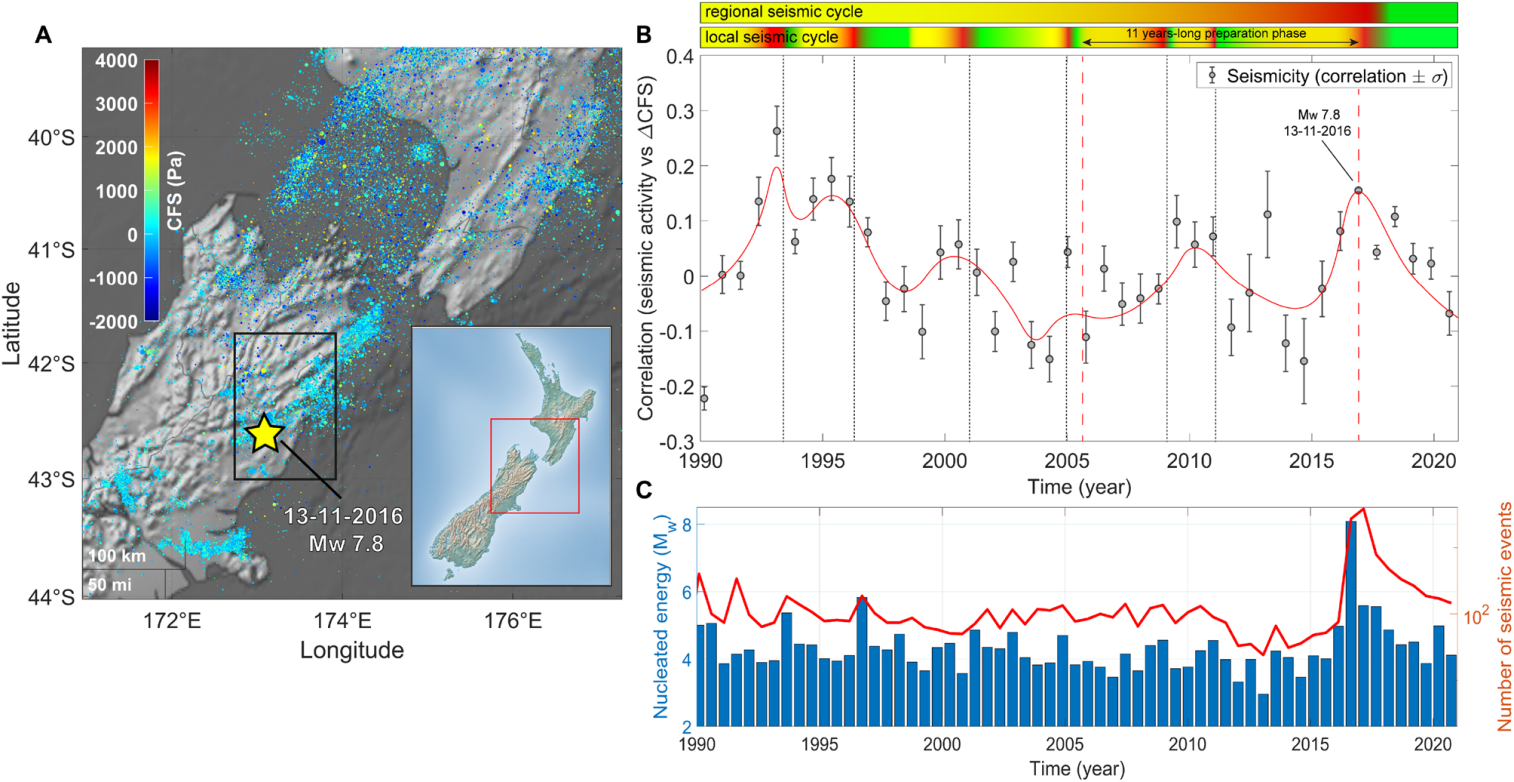
(**A**) $\Delta$CFS map for seismicity in the Christchurch region, South Island, New Zealand, GeoNet Catalog, 1990−2021, M$_{L} \ge$ 2.5. The map is realized using the functions “geoscatter” and “geobasemap” in the Matlab environment^63^. (**B**) Correlation $\rho$ between $\Delta$CFS and seismicity in the Kaikoura District, South Island, New Zealand (latitude 42.1°−43.1° S and longitude 172.7°−173.9° E, depth 0−33 km, M$_{L}$ $\ge$ 2.5) between 1990 and 2021. The red line is a weighted smoothing spline. Two horizontal color bars above the scatter-plot show nested “seismic cycles” whose phases are highlighted by different colors (green for the post-seismic phase, the interseismic one is in yellow and the coseismic phase is in red). (**C**) Histogram of energy nucleated by earthquakes with M$_{L}$ $\ge$ 2.5 (blue bars) and plot of the number of recorded seismic events (red line).

New Zealand is prone to large seismic events because of its extremely active tectonic environment. In particular, in South Island the collision of the Pacific and the Australian plates is responsible for the growth of the Southern Alps. Significant faulting is also present in the region of Canterbury and Christchurch, able to nucleate large quakes (e.g., M$_{w}$ 7.8, Murchison, 1929; M$_{w}$ 7.1, Darfield, 2010). We perform correlation analysis between tidal stress and seismic nucleation rate in two separate regions in South Island (Figs. 2 and 3):

- Fiordland Region, only considering events localized within latitude 45.3°−46.2° S and longitude 166.0°−167.0° E at depth 0−33 km and with M$_{L}$ $\ge$ 2.5 between 1990 and 2021. The region has been shaken by the 10/08/1993 M$_{w}$ 6.7 earthquake and by the 15/07/2009 M$_{w}$ 7.8 Great Fiordland event^16^. Our work reveals positive correlations between $\Delta$CFS and nucleated energy in correspondence of moderate and large magnitude seismicity. Positive correlations are also recorded concerning seismicity triggered by major events occurred in near regions (e.g., M$_{w}$ 7.2, 22/08/2003). Negative values of $\rho$ are measured during locking periods, which is in good agreement with our expectations. Compare with Fig. 2.
- Kaikoura region, between latitude 42.1°−43.1° S and longitude 172.7°−173.9° E. This territory hosted one of the most violent earthquakes in the history of New Zealand and it is of broad geophysical and tectonic interest because of the local intricacy of the Marlborough Fault System. The M$_{w}$ 7.8 Kaikoura earthquake, which shook South Island on 13$^{th}$ November 2016, was indeed associated with a complex array of surface ruptures that involved about 20$-$30 different active faults^17^. Correlation analysis shows that seismicity tends to occur at positive $\rho$ values in this region (Fig. 3). Especially, peaks are recorded in 1994 and 1997, when diffuse moderate magnitude events happened, and it is followed by an about nine-years-long decrease. In 2010$-$2011 a new bump is observed due to the seismicity triggered by the Christchurch and Darfield earthquakes, that happened just outside the selected area. The M$_{w}$ 7.8 event occurred at $\rho \sim$ 0.16 after about ten years featured by a progressively increasing trend which abruptly accelerated its raising two years early. Aftershocks occurrence is found to less and less correlate with tidal stress as time goes, as expected.

### Central Italy

We focus our attention on the segment of the Central Italian Apennines located in between the towns of L’Aquila and Gubbio. The first series of large magnitude earthquakes listed in the INGV seismic catalog took place from 26$^{th}$ September 1997 until the spring of the following year. The largest event occurred on 26$^{th}$ September with M$_{w}$ 6.0^18^. Within some months, the preceding seismic activity was re-established. For years, Central Italy has been hit by isolated swarms whose quakes were featured by M$_{L} < 4.0$, until December 2008, when seismicity experienced a swift increase both in magnitude and in frequency. It culminated with the M$_{w}$ 6.3 L’Aquila earthquake^19^, nucleated on 6$^{th}$ April 2009, which was followed by a three-years-lasting sequence which spread to north. On 24$^{th}$ August 2016 a quake of M$_{w}$ 6.0^20^ occurred in between the villages of Accumuli and Norcia. Aftershocks overflowed onto a wide territory spanning four Italian regions. Seismic activity reached a new peak on 26$^{th}$ October, when two earthquakes (M$_{w}$ 5.4 and M$_{w}$ 5.9) shook Visso and its countryside. The seismic rate kept high with also a further increase on 30$^{th}$ October, after the mainshock of M$_{w}$ 6.5^21^, whose epicenter was localized 4 km NE of Norcia. Earthquakes of significant magnitude were recorded for months, among them, a M$_{w}$ 5.5 event hit Campotosto on 18$^{th}$ January 2017. The Amatrice-Visso-Norcia (AVN) seismic sequence is still ongoing.

Our analysis is performed into two different zones: within latitude 42.7°−43.1° N and longitude 12.9°−13.3° E (Valnerina area) and at latitude 42.2°−42.8° N and longitude 13.1°−13.6° E (Laga Mountains and the North L’Aquila province) between 1985 and 2021.

**Figure 4:**
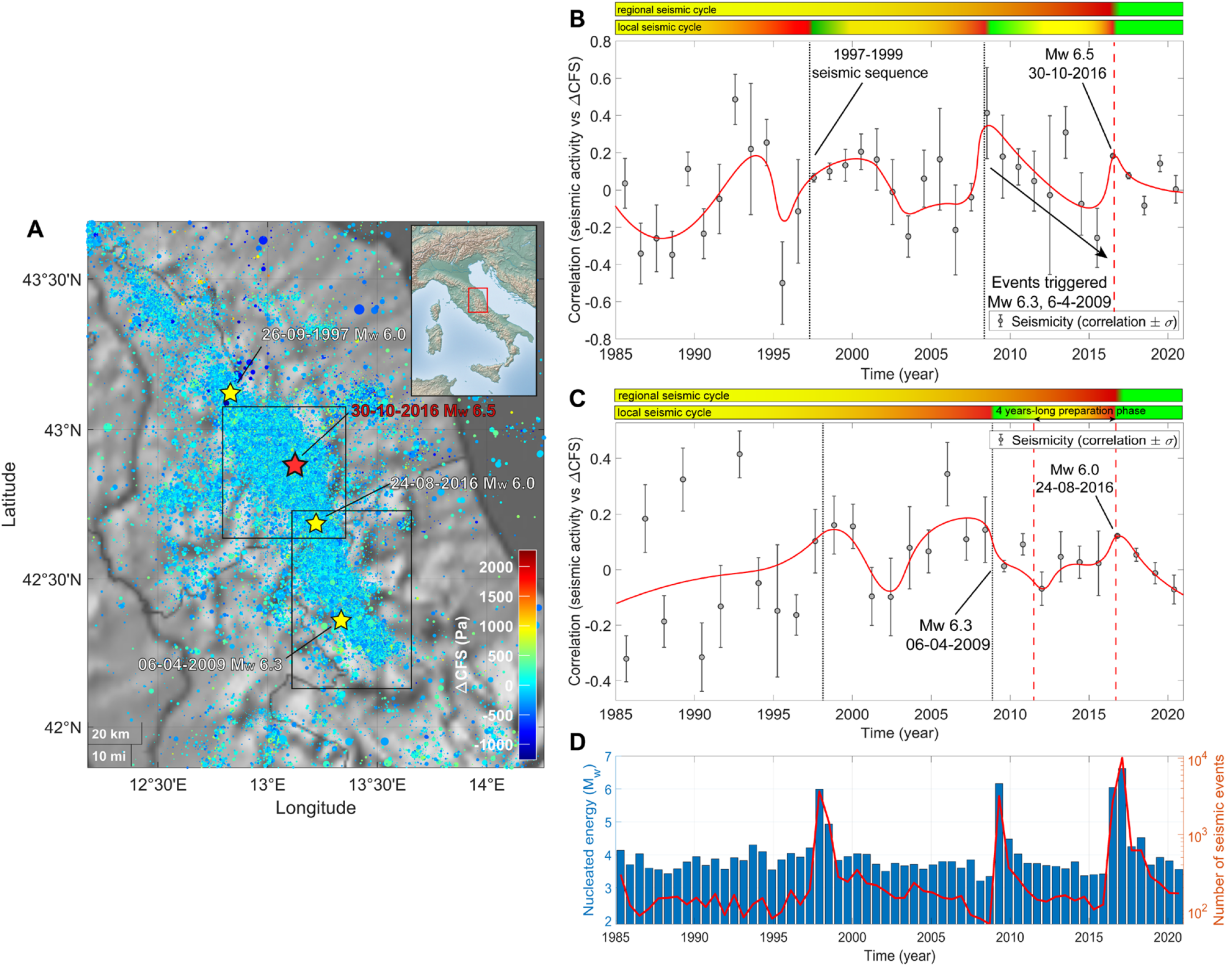
(**A**) $\Delta$CFS map for seismicity in Central Italy, INGV Catalog, 1985−2021, M$_{L} \ge$ 2.0. The map is realized using the functions “geoscatter” and “geobasemap” in the Matlab environment^63^. (**B**) Correlation $\rho$ between $\Delta$CFS and seismicity in the Valnerina area between 1985 and 2021. The plot is realized by taking into account earthquakes happened at latitude 42.7°−43.1° N and longitude 12.9°−13.3° E. Only 5−15 km deep seismic events are considered, M$_{L}$ $\ge$ 2.0. The red line is a weighted smoothing spline. Two horizontal color bars above the scatter plot show nested “seismic cycles” whose phases are highlighted by different colors (green for the postseismic phase, the interseismic one is in yellow and the coseismic phase is in red). (**C**) Correlation $\rho$ between $\Delta$CFS and seismicity in Central Italy, Monti della Laga and North L’Aquila province, between 1985 and 2021. The plot is realized by taking into account earthquakes happened at latitude 42.2°−42.8° N and longitude 13.1°−13.6° E. Only 0−15 km deep seismic events are considered. (**D**) Histogram of energy nucleated by earthquakes with ML $\ge$ 2.0 (blue bars) and plot of the number of recorded seismic events (red line).

Seismicity in the Valnerina area shows increased $\rho$ (compare with the right scatter plots in Fig. 4) before the 1997 Colfiorito seismic sequence; a new peak is reached nearby L’Aquila earthquake, then a decreasing trend develops for about seven years, till the beginning of the AVN sequence. This behavior suggests that remote triggering occurred in the Norcia area because of the crustal destabilization provided by the L’Aquila sequence. In the southernmost situated region, progressive gain of correlation is less affected by the 1997−1998 seismic activity, continuing its growth until 2008, when a drop is observed. A possible explanation is that preslip and foreshocks partially released stress before L’Aquila event. This possibility is compatible with^22^ and other evidences such as the intense foreshock activity. Our interpretation of the dynamics featuring the Central Italy seismic activity is that a 20 years-long destabilization characterized by swarms, small earthquakes with few events of relevant magnitude culminated with the 6$^{th}$ April 2009 M$_{w}$ 6.3 earthquake, which likely accelerated the “seismological clock” in the northernmost segment of the activated fault system. The final output of this process was the M$_{w}$ 6.5 Norcia mainshock, which closed a “regional seismic cycle” which had been lasting since the Great Cascia-Norcia earthquake occurred on 14$^{th}$ January 1703 (M$_{w}$ 6.8)^23^.

## Discussion

### Correlation analysis and precursors

So far we have portrayed the main features of local response of fault systems and seismicity to tidal stress modulations. We shed a light on how correlation between tides and nucleated seismic energy depends on the instability of the geological structure showing recurrent patterns revealing impending seismicity. Our analysis is also sensible to foreshock activity, preslip and seismic quiescence. However, many questions still remain unsolved concerning tidal triggering of earthquakes. How long do preseismic trends last? What is the relation with pending events and their magnitudes? Is an application to seismic hazard possible? It is likely that a simple correlation analysis will never be able to provide reliable information about the occurrence of future seismic events more than other “precursors” (e.g., changes in the velocity of seismic waves, geodetic, seismological, geo-electric and hydro-geochemical monitoring). Therefore, it is improbable that it will ever be possible to define alert levels based on this method. In support of this, several observations can already be enumerated: the first concerns the extreme variability of the correlation values and associated uncertainties, in any case rather low, recorded during seismic dynamics; the second regards the marked dependence of the result on the quality and quantity of the seismological data. Furthermore, the calculation of the correlation should be carried out by normalizing the stress values as a function of the location of hypocenters. Finally, it would be necessary to include in the model several sources of disturbance that can alter the output.

**Figure 5:**
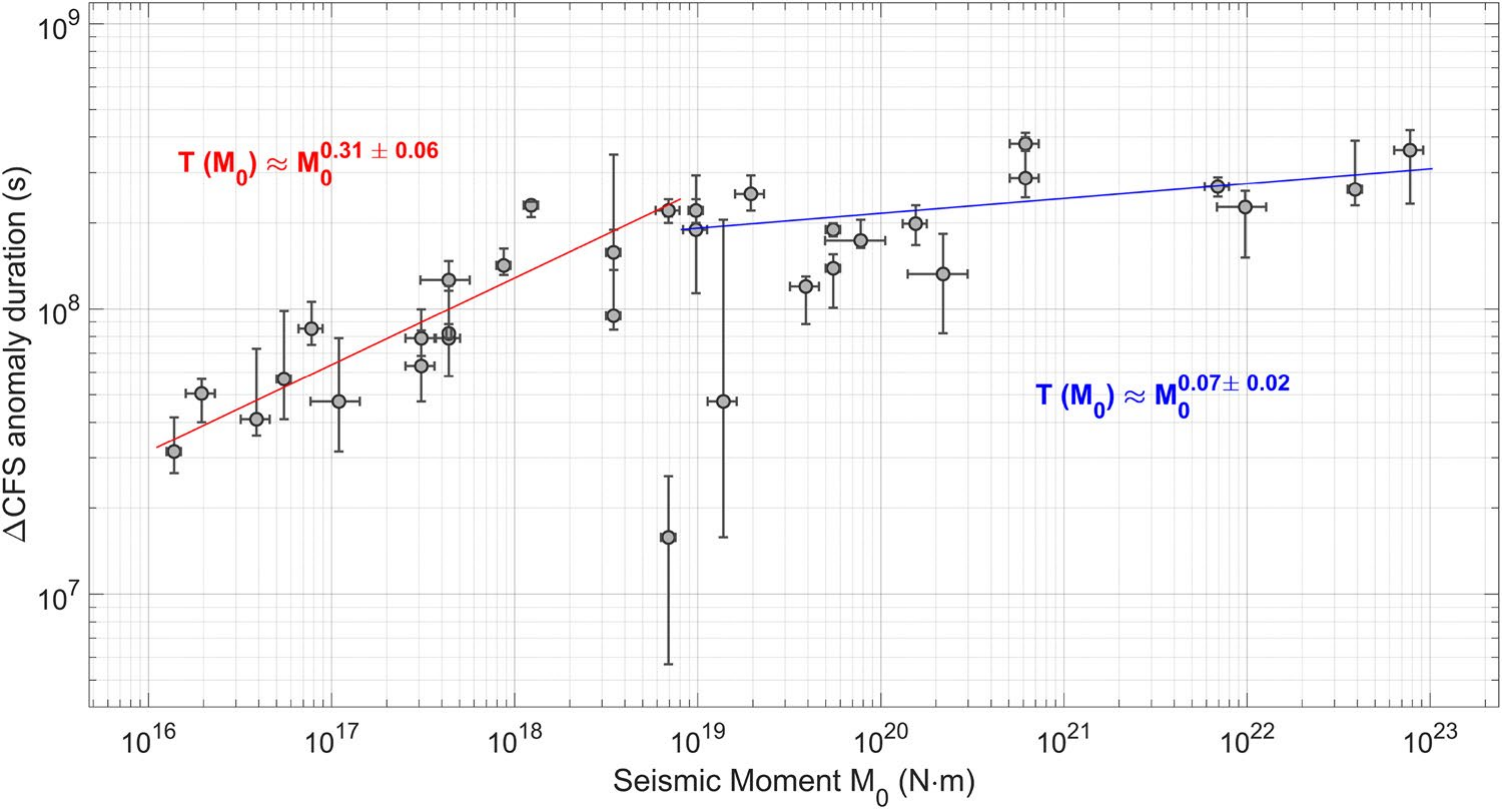
Preparatory phases are observed before thirty-five large and intermediate (M$_{w} \gtrsim 4.5$) shallow (depth $\le 50$ km) earthquakes all over the world. Each measure with error-bar corresponds to a seismic sequence. The duration of the anomalies is empirically related to the seismic moment of the impending mainshock through $T \approx M_{0}^{0.3}$ for M$_{w} \lesssim$ 6.5, while the scaling relation becomes $T \approx M_{0}^{0.1}$ for M$_{w} \gtrsim$ 6.5, according to the best-fits, represented, respectively, by the continuous red and blue lines. Our results support the hypothesis that seismic regions are over-stressed for months up to 15−years−long periods before main failure. However, variability is large, although there is a positive correlation between duration of increasing seismic responsiveness to stress perturbations and magnitude of impending earthquakes. A better estimation of the scaling exponents should be done including additional low magnitude ($\lesssim$ 4.5) seismic events. Fit R$^{2}_{\lesssim 6.5}$ = 0.94, R$^{2}_{\gtrsim 6.5}$ = 0.83.

Nonetheless, the response of seismic activity to tidal stress may be a useful tool for understanding the physics of earthquakes. In this regard, a lot of processes occurring over long time scales still remain elusive and cannot be analysed with the usual seismological techniques and, sometimes, not even with geodetic ones. In our analysis, correlation time series between $\Delta$CFS and seismic nucleation rate are calculated for several seismic time series, among them three cases have been discussed in the previous section. Two different patterns before major sequences are highlighted:

- A progressive increase is recorded in about 60$\%$, 22 cases, in our statistics made up of 35 seismic sequences, whose duration is highly variable (compare with Fig. 5). The correlation reaches its peak just before the seismic activity starts, then the value falls. From a seismological point of view, regions featured by this trend show seismic quiescence^24^ or no significant changes in the seismic rate before the mainshock. This behavior is noticed, for instance, in the area located surrounding Accumuli before the AVN sequence, compatible with the results in^25^, before the Colfiorito seismic sequence in 1997$-$1998, also coherent with^26^. A growth of $\rho$ is also recorded before the Ridgecrest earthquake (compare with Fig. 1), in agreement with^15^.
- A continuous decrease of correlation followed by a positive jump just before or during the seismic crisis is detected in 20$\%$ of cases. The drop of $\rho$ is associated with an increasing seismic activity (e.g., this is the situation occurred before L’Aquila earthquake in 2009 and the 2018 Molise sequence) or preceded by intense seismic activity, such as before the 2015 M$_{w}$ 6.5 Lefkada earthquake^27^.

We also find cases (about 20$\%$) in which no significant change of $\rho$ is identified before large earthquakes, for instance during the 2000 South Iceland seismic sequence.

Speaking of duration, there is evidence^28^ that the crust is in a critical state only under certain conditions often associated with imminent and widespread seismicity. The development of a critical state is what is believed to underlie the intense long-range spatial correlations and the surprising capacity of faults to react to tiny stress sources such as tides. However, how such critical states are formed and how long they take to emerge is a question that cannot be answered yet.

The duration of over-stressing phases featured by the growth of a critical state is one of the crucial issues that could be investigated with the method described in the present paper.

Even though a detailed measure of the duration of preparation phases remains elusive, a coarse-grained estimation is possible. The procedure we used is described in the section devoted to methods.

Our results are shown in Fig. 5. The number of data is limited since we have studied only thirty-five sequences and certainly a better understanding of the processes occurring before major events can be achieved by expanding our statistics, for instance including additional low magnitude earthquakes. Anyway, data are enough to assert that the duration T of the $\Delta$CFS preparatory phase is positively correlated with the magnitude of the impending mainshock. A linear fit covering the database all at once returns T $\approx$ M$_{0}^{0.23 \pm 0.09}$ (R$^{2}$ = 0.51). Its quality is lousy; moreover, there are no specific reasons or theoretical models accounting for this result. The situation becomes quite different if a threshold seismic moment is set at M$_{0}^{th} \approx$ 10$^{19}$ N m, corresponding to a moment magnitude M$_{w}^{th} \approx$ 6.5, so that it is assumed that two different scaling behaviors exist below and above this value. The output of the double weighted linear fit in log-log scale is

1 $$\begin{aligned} {{\left\{ \begin{array}{ll} \displaystyle T \approx M_{0}^{0.31 \pm 0.05},~~~M_{0}\le M_{0}^{th},~~~R^{2} = 0.94\\ \displaystyle T \approx M_{0}^{0.07 \pm 0.02},~~~M_{0}\ge M_{0}^{th},~~~R^{2} = 0.83 \end{array}\right. }} \end{aligned}$$

This result is in good agreement with the works concerning the preparatory phase of giant events (e.g.,^29,30^) and compatible with suggested positive feedback mechanisms in earthquake occurrence^31^.

### A possible theoretical interpretation

The first exponent, $0.31 \pm 0.05$, is compatible with the scaling behavior of the nucleation phase suggested in^32^ for single seismic events. In^32^ is proposed that the duration of nucleation phase is directly proportional to the size of the earthquake (T $\approx$ M$_{0}^{0.33}$) and associated with the time required for the slip-weakening displacement to occur. This analogy suggests that the physical mechanism that heralds the coseismic failure at very short time scales (order of magnitude 10$^{-5} -$10$^{1}$ s), may be similar to the preparation processes of seismic sequences, taking place over longer times, usually months or years, identified in our work. The second exponent turns out to be more difficult to explain. It does not appear to be connected with the scaling laws of the nucleation phase. We propose that the lower value of the second exponent can be understood on the light of different mechanisms generating seismic events with $M_{w} \ge$ 7. Several of these earthquakes are caused by the cascade activation of faults via direct triggering due to stress transfer across crustal volumes. Therefore, a shorter duration of the preparation phase of large magnitude earthquakes can be explained advocating a parallel preparation process in neighboring faults that eventually fall into simultaneous activation.

In summary, the duration of increasing trends we observe in the aforementioned correlation time series might be interpreted in the light of diffuse nucleation phases throughout the crust.

However, only thirty-five seismic sequences are analysed and data are affected by large uncertainties that cannot be neglected, so further theoretical considerations can be useful to check the soundness of our results.

The theory of critical systems states that, given an external perturbing field, *H*, for the “magnetization” a system acquires due to *H*, *m*, i.e., the variation of free energy of the system with respect to the amplitude of the perturbation, the following scaling relation holds^33^

2 $$\begin{aligned} m \propto H^{1/3} \end{aligned}$$

in the mean-field regime. By identifying the perturbing field with the variation of Coulomb failure stress due to tides, $H = \Delta CFS$, and being the fault internal stress state the natural candidate for the “magnetization” of fault systems, then the following interpretation could be given: the differential stress progressively grows as time goes due to tectonic strain and additional deformation sources; therefore, called *T* the time has passed since the critical state started emerging, the seismic rate $\sum _{i}^{V_{f}}\int _{A_{i}}\mu \langle \delta u_{i}\rangle dS$, where the sum is done over the faults beside the failure volume $V_{f}$, also tends to rise

3 $$\begin{aligned} T \propto \left( \sigma - \sigma _{breaking}\right) ^{-1} \propto \sum _{i}^{V_{f}}\int _{A_{i}} \mu \langle \delta u_{i}\rangle dS = R_{(V_{f})} \end{aligned}$$

where the local stress $\sigma$ becomes closer and closer to the critical point $\sigma _{breaking}$, $\mu$ is the shear modulus and $\langle \delta u_{i}\rangle$ is the mean slip of the fault $A_{i}$. In addition, the seismic activity $R_{(V_{f})}$ is also positively correlated with the additional stress

4 $$\begin{aligned} R_{(V_{f})} \propto \langle \Delta CFS\rangle \propto \sum _{i}^{V_{f}}\int _{A_{i}} dS \propto \int _{A} \mu \langle \delta u\rangle dS = M_{0} \end{aligned}$$

where $\langle \Delta CFS\rangle$ is the average additional Coulomb failure stress at which earthquakes occur and M$_{0}$ is the moment of the forthcoming mainshock nucleated by the fault plane *A*. Hence, it is reasonable that the duration of the preparation phase of major seismic sequences and the seismic moment of the impending mainshock be identified as critical parameters of fault systems and be directly proportional to each other according to the following power-law

5 $$\begin{aligned} T \propto M_{0}^{1/3} \end{aligned}$$

### The role of seismic clustering

The correlation between tidal stress and nucleated seismic energy is affected by several factors that should be taken into account for its correct interpretation. The most important are:

- If full catalogs are used, i.e., no declustering is performed, correlations depend on both the frequencies of stress modulation and the clustering correlation time. Therefore, if the main stress modulation period, $\tau _{stress}$, is equal or smaller than the duration of the seismic cluster ($\tau _{cluster}$), then the measured correlation value, $\rho _{m}$, is exponentially suppressed with respect to the real one $\rho _{r}$:
  6 $$\begin{aligned} \rho _{m} \approx \rho _{r} {}^{-\Lambda \frac{\tau _{cluster}}{\tau _{stress}}}, \end{aligned}$$
  where $\Lambda$ is a positive and real suitable fit parameter. Changes in the size of clusters due to long-lasting seismic activity is one of the reasons why correlations tend to vanish after major seismic events. Further investigations are needed in order to understand whether spurious suppression in correlations can overshadow possible increase due to new impending earthquakes. However, previous observations also imply that variations in statistical properties of clusters are significant during the different stages of seismic dynamics and, which is more important, are also detected during the preseismic phase, which is compatible with the results of several research works, e.g.,^34–36^. A clear evidence of this effect is given in the fourth and fifth columns of Table 1.
- Seismic clusters play a key role in triggering new events. Declustering almost completely suppresses correlation values. This is the reason why in the previous section only full catalogs are analysed, while in Table 1 we also list our outputs for declustered seismic time series. Moreover, stress transfer due to internal self-organized processes can be responsible for negative values of correlations suggesting that crustal volumes are going to recover stability, so that seismicity is less responsive to tidal stress and its occurrence rate is not affected by unfavourable conditions, while failures may be promoted with larger probability through highly non-linear, out-of-phase processes.
- An other possible mechanism may contribute to the aforementioned results depends on the granular behavior of the brittle crust. Fault zones can be sketched as thoroughly fractured and highly pressurized rocks drenched of water. Now, a water-saturated granular soil reacts in a peculiar way to stress pulses, which is quite different from that of a solid material. Since liquids are extremely reactive to pressure, water is immediately squeezed outwards from the fault zone as the incremental tidal stress compacts rocks and it usually percolates into an aquifer. This kind of phenomena is largely documented both for tidal and seasonal loading (e.g.,^37^) and for seismic stress transfer within the fault zone. Suppose a granular medium (i.e., fractured rocks) is immersed in water at initial pressure $P_{0}$. The system is at rest and the volume fraction occupied by the solid material is $f_{0}$. Then a sudden incremental stress is added (i.e., tidal loading), so that the applied pressure becomes $P_{0} + \delta P$. For the sake of simplicity, we can assume that stress transfer occurs only along the direction *x* orthogonal to the fault plane. If the deformation generated by the stress variation is small (which is verified for tidal stress since it is $\sim$ 10$^{-4}$ tectonic stress) the linearized constitutive equation is
  7 $$\begin{aligned} P(f) = P_{0} + E(f-f_{0}) \end{aligned}$$
  where *E* stands for the elastic modulus; so the granular pressure satisfies the well-known Fick’s diffusion equation
  8 $$\begin{aligned} \frac{\partial P}{\partial t} = \frac{\kappa (f_{0})f_{0}E}{\eta } \frac{\partial ^{2} P}{\partial x^{2}} \end{aligned}$$
  where $\kappa (f_{0})$ is the permeability of the granular medium and $\eta$ represents the viscosity of liquids. The response to stress depends on the diffusion coefficient, which in the case of fault zones is extremely variable, so that the characteristic time scale is^38^
  9 $$\begin{aligned} \tau _{char} \approx \frac{W^{2}\eta }{\kappa (f_{0})E} \le 10^{6}~s \end{aligned}$$
  where *W* is the width of the fault zone ($\sim$ 10$^{-1}$ $-$10$^{2}$ m^39^), $\eta _{water} \sim$ 10$^{-4} -$ 10$^{-3}$ Pa s (100−300°C, 1−100 MPa), $\kappa \sim$ 10$^{-17}$ - 10$^{-14}$ m$^{2}$^40^ and $E \sim$ 10$^{10} -$10$^{11}$ Pa^41^. Since $\tau _{char}$ is larger than the diurnal and semi-diurnal tidal periods ($\sim$ 10$^{4}$ s), then the effective additional stress induced by high frequency tidal perturbations can be smaller than expected.

**Table 1:** Correlation values between tidal $\Delta$CFS and nucleated energy concerning sixteen full-catalog and declustered one-years-long seismic time series in correspondence of moderate and large seismic sequences. The time series starts six months before the mainshock and stops six months later. Only shallow (depth $\le$ 50 km) events occurring within the future epicentral area (rectangular regions hit by increased seismic activity during the impending seismic sequence) above the completeness magnitude are considered in this analysis. The fourth column shows our results for full seismic catalogs ($\rho _{0.5d}$), while in the fifth one correlations are calculated for simulated tidal stress in which semi-diurnal and diurnal harmonics are removed ($\rho _{14d}$). The last column contains the outputs for Uhrhammer-declustered^42^ catalogs ($\rho _{0.5d}^{(DECL)}$). Declustered time series do not provide useful information about tidal triggering of earthquakes, since the correlation values are always compatible with zero. These results strongly suggest that the statistical properties of clustering change as a function of the stability state of crustal volumes. This is obvious after large earthquakes occur, as aftershocks prove, but how earthquake clusters are affected by local spatial and temporal stress patterns is still a puzzle. For the sake of readability, in the present work, except for this table, only results for $\rho _{0.5d} = \rho$ time series are shown.

**Place**	**Earthquake**	**M**$_{w}$	$\rho _{0.5d} (\sigma _{\rho })$	$\rho _{14d} (\sigma _{\rho })$	$\rho _{0.5d}^{(DECL)} (\sigma _{\rho })$
Ormond	10/06/1993	6.7	0.12 (0.02)	**0.18 (0.03)**	− 0.02 (0.10)
Colfiorito	26/07/1997	5.6	0.07 (0.01)	**0.09 (0.01)**	0.00 (0.04)
Lithakia	18/11/1997	6.6	**0.08 (0.03)**	0.05 (0.02)	− 0.01 (0.02)
South Iceland	29/05/2000	6.5	**0.10 (0.01)**	0.00 (0.01)	0.00 (0.08)
Molise	31/10/2002	5.7	0.06 (0.01)	**0.15 (0.03)**	0.04 (0.12)
L’ Aquila	06/04/2009	6.3	0.09 (0.01)	**0.42 (0.08)**	0.03 (0.08)
Fiordland	15/07/2009	7.8	0.07 (0.01)	**0.39 (0.02)**	0.03 (0.07)
Tohoku	11/03/2011	9.0	**0.19 (0.03)**	0.13 (0.05)	0.04 (0.10)
Emilia	20/05/2012	5.9	0.05 (0.01)	**0.07 (0.01)**	− 0.03 (0.12)
Lefkada	26/01/2014	6.1	0.13 (0.02)	**0.14 (0.03)**	0.01 (0.10)
Amatrice	24/08/2016	6.0	0.06 (0.01)	**0.19 (0.02)**	− 0.02 (0.06)
Kaikoura	13/11/2016	7.8	0.16 (0.01)	**0.37 (0.02)**	0.02 (0.08)
Molise	16/08/2018	5.1	**0.21 (0.02)**	**0.21 (0.02)**	− 0.02 (0.17)
Lithakia	25/10/2018	6.8	**0.14 (0.02)**	0.08 (0.02)	0.01 (0.06)
Ridgecrest	04/07/2019	6.4	**0.24 (0.01)**	0.12 (0.04)	0.07 (0.08)
Thessaly	03/03/2021	6.3	**0.29 (0.02)**	0.26 (0.01)	0.05 (0.06)

### General features of the response of seismicity to additional stress

**Figure 6:**
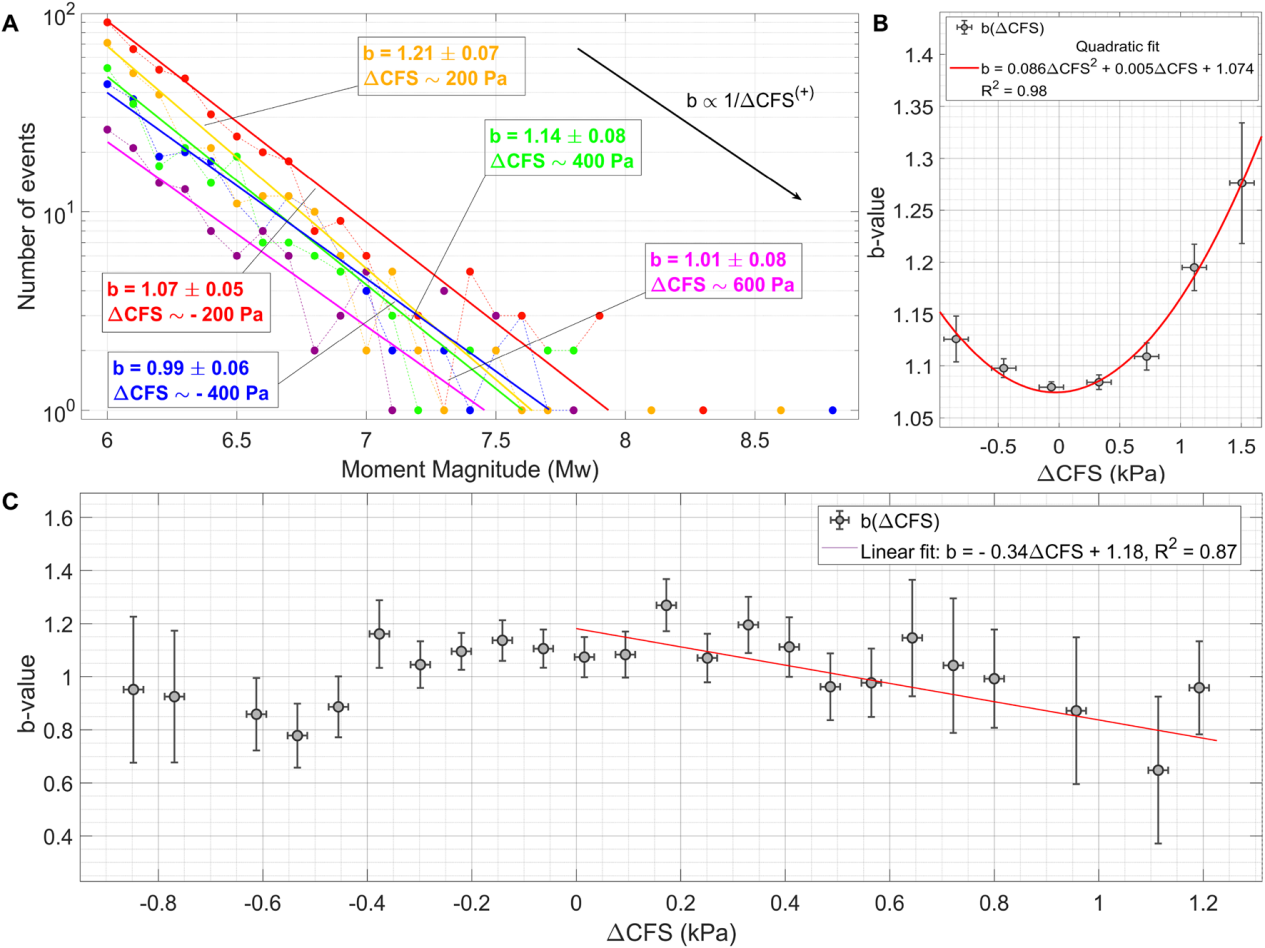
(**A,C**) *b* is inversely correlated to the intensity of the tidal Coulomb failure stress for large magnitude seismic events. The plot is realized by analysing worldwide seismic events in USGS catalog between 2001 and 2021, M$_{w} \ge$ 6.0 (M$_{wc} \approx$ 5). (**B**) *b*($\Delta$CFS) is well fitted (R$^{2}$ = 0.98) by a parabolic function if only shallow worldwide M$_{w} \ge$ 4.6 seismic events are included. The plot is realized by analysing seismic events in USGS catalog between 2001 and 2021, depth $\le$ 50 km. If compared to the scatter-plot in the lower part of this figure, the difference of the trends strongly suggests that low magnitude shallow earthquakes are more sensitive to tidal stress with respect to larger ones. This conclusion is consistent with Fig. 7.

Incremental stress sources modify the seismic nucleation rate R(t) and the dissipated moment by lowering the activation energy needed to break up asperities. This means that perturbations enhanced by the additional stress inside rock volumes are more likely to be transmitted to nearby asperities and to propagate. In particular, it is well known^43^ that the exponent of the Gutenberg-Richter law is inversely proportional to the intensity of the perturbing field at equilibrium, so that for more intense tidal stress larger magnitudes are slightly more probable. It is exactly what is shown in Fig. 6. The plot is realized by analysing worldwide seismic events in USGS catalog between 2001 and 2021, M$_{w} \ge$ 6.0. However, a curious phenomenon is noticed if also intermediate magnitude events are included in the analysis: *b*($\Delta$CFS) appears to increase with stress, in our case it is well fitted by a parabolic function. Compare with Fig. 6B; the plot is realized by studying worldwide seismicity (USGS catalog) between 2001 and 2021, depth $\le$ 50 km and M$_{w} \ge$ 4.6. That differences in *b*-values were less clear for events of M$_{w} < 6.5$, has been already reported in scientific literature, but this effect has not been examined any further. In this regard, it was suggested^44^ that catalogs include events with various focal mechanisms in different tectonic environments and hence the mixing of events with a wide range of *b*-values could be the reason of this anomaly. In fact, a comparison of Fig. 6B with Fig. 6C strongly suggests that low magnitude shallow earthquakes are more sensitive to tidal triggering with respect to larger ones. In order to better understand what happens to the *b* value for M$_{w} < 6.5$, we also investigate “more homogeneous catalogs” by making careful selection of focal mechanisms, but no significant changes are observed with respect to the previous output. A simple investigation of the average value of the tidal Coulomb failure stress at which earthquakes occur globally confirms what has been hypothesized so far: seismicity becomes more and more responsive to tidal stress as magnitude decreases, which is consistent with the results discussed in^45^.

**Figure 7:**
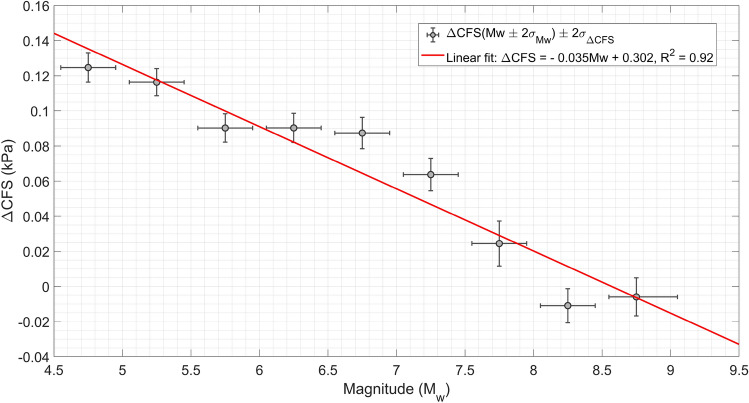
Moderate seismicity is more strongly triggered by solid Earth tides than larger earthquakes. Stress perturbations directly affect small unstable faults more likely than larger ones, while fracture propagation over large patches is almost completely controlled by their internal state of stress. The plot is obtained by averaging the tidal Coulomb Failure stress values over groups of earthquakes with different moment magnitudes. The figure is realized by analysing worldwide seismic events listed in the USGS catalog between 2001 and 2021, M$_{w} \ge$ 4.5.

Our results are summarized in Fig. 7, which is obtained by averaging tidal Coulomb failure stress $\Delta CFS$ over groups of earthquakes with different moment magnitudes. The plot is realized analyzing worldwide seismic events listed in the USGS catalog between 2001 and 2021, M$_{w} \ge$ 4.5. The plot shows that events of magnitude larger than 8 seem not to be significantly affected by tidal stress. This might appear contrary to the results described in the previous section. We had observed that the maximum values of $\rho$ for local seismicity is recorded in correspondence with the maximum stress state of the fault. Anyway, it does not mean that the single seismic event, i.e., the mainshock, is more affected by the additional stress, but only that, as a whole, this is true on average. The crux of the matter is that fault heterogeneity plays a key role on large scale control of fracturing^46^. While this effect is limited at small spatial scales, where local stress dominates fracture dynamics, large earthquakes are rare events whose probability of occurrence during periods of elevated additional stress only derives from the occurrence of the average low-magnitude seismicity. Then, it is a “second order” effect, weaker than for small and intermediate seismic activity, which can be assumed to happen regardless of long range interactions among fault systems.

## Methods

### Tidal stress calculation

Tidal stress perturbations are evaluated at the timing of each earthquake taking into account the hypocentral coordinates and the spatial orientation of the fault plane, with the same method used in a previous paper^27^. We describe it briefly. Starting from the components of displacement produced by tidal perturbations W(r, $\theta$, $\phi$) in spherical coordinates

10 $$\begin{aligned} {\left\{ \begin{array}{ll} u_{r}=\dfrac{h_{2}(r)}{g(r)} W(r,\theta ,\phi ) \\ u_{\theta }= \dfrac{l_{2}(r)}{g(r)} \dfrac{\partial W(r,\theta ,\phi )}{\partial \theta }\\ u_{\phi }= \dfrac{l_{2}(r)}{g(r)\sin \theta } \dfrac{\partial W(r,\theta ,\phi )}{\partial \phi }\\ \end{array}\right. } \end{aligned}$$

where *g*(*r*) is the gravitational constant as a function of depth (R-r), with R the radius of the Earth, strain is obtained by derivation, so that stress components are

11 $$\begin{aligned} {\left\{ \begin{array}{ll} \sigma _{\theta \theta }(r)= \lambda (r)\left( \varepsilon _{rr} + \varepsilon _{\phi \phi } + \varepsilon _{\theta \theta }\right) + 2\mu (r)\varepsilon _{\theta \theta }\\ \sigma _{\phi \phi }(r)= \lambda (r)\left( \varepsilon _{rr} + \varepsilon _{\phi \phi } + \varepsilon _{\theta \theta }\right) + 2\mu (r)\varepsilon _{\phi \phi }\\ \sigma _{\theta \phi }(r)= \mu (r)\varepsilon _{\theta \phi }\\ \end{array}\right. } \end{aligned}$$

where the Lame’s coefficients can be obtained starting from the speed of the seismic P and S waves as a function of depth (using PREM data^47^) as follows

12 $$\begin{aligned} {\left\{ \begin{array}{ll} \lambda (r)= d(r)\left( v^{2}_{P}(r) - 2v^{2}_{S}(r) \right) \\ \mu (r)= d(r)v^{2}_{S}(r)\\ \end{array}\right. } \end{aligned}$$

d(r) is the density of internal Earth at depth R-r. At last, the spatial orientation of faults provides information about the tectonic stress tensor. Given the strike $\alpha$ and the dip angle $\delta$ of the seismological source, the tangential stress^48^ is given by

13 $$\begin{aligned} \sigma _{\alpha }^{(\pm )} = \sigma _{\theta \theta }(r) \cos ^{2}\alpha + \sigma _{\phi \phi }(r) \sin ^{2}\alpha \pm 2\sigma _{\theta \phi }(r) \sin \alpha \cos \alpha \end{aligned}$$

and the principal stress components are

14 $$\begin{aligned} {\left\{ \begin{array}{ll} \sigma _{s}= \sigma _{\alpha }^{(+)} \sin \delta \cos \delta \\ \sigma _{n}= \sigma _{\alpha }^{(+)} \sin ^{2}\delta \\ \sigma _{c}= \dfrac{1}{3}\left( \sigma _{\alpha }^{(+)} + \sigma _{\alpha }^{(-)}\right) \\ \end{array}\right. } \end{aligned}$$

respectively the shear stress, the normal stress and the confining stress. $h_{2}(r)$ and $l_{2}(r)$ can be calculated by integrating with the fourth order Runge−Kutta method a system of six coupled ordinary differential equations starting from a set of suitable boundary conditions^49^.

The angle of dip and the angle of strike are measured via focal mechanisms in the case of large earthquakes, while the mean value of available dip and strike angles is associated to seismic events whose moment tensor is not known. The uncertainties of the dip and strike angles are obtained from the rule for the mean values. Ocean loading can induce stress up to 100 kPa that is much larger than the stress due to solid tides (0.1−1 kPa); nevertheless, it is generated locally and usually focused over small surfaced with some exceptions, so that its impact on earthquakes nucleation is limited^50^. In practice, the main contribution of ocean tides derives, unlike solid tides, from vertical stress^51^

15 $$\begin{aligned} \sigma _{zz}= - dgh \end{aligned}$$

which is buffered as the hypocentral depth increases, where *h* is the amplitude of the tide and *d* is the density of the sea water. The radial stress spreads horizontally through the Poisson’s coefficient, therefore, if we assume that the vertical stress acts symmetrically $\sigma _{xx}$=$\sigma _{yy}$= $\dfrac{\nu }{1-\nu }\sigma _{zz}$.

The predicted tidal height *h* is provided by the NAO.99b software^52^.

However, in our analysis we utilize the variation of Coulomb Failure Stress $\Delta$CFS^53^

16 $$\begin{aligned} \Delta CFS = \sigma _{s} + \mu \sigma _{n} \end{aligned}$$

where $\mu \sim 0.4-0.8$, which has a straightforward physical interpretation: positive $\Delta$CFS is associated with encouraged seismicity, while negative values produce shadow effects suppressing slip^54^.

### Correlation and error analysis

Once tidal stress is available and regions of geophysical interest are selected, we perform the following steps:

- Estimation of the completeness magnitude.
- Local magnitudes M$_{L}$ are converted into moment magnitudes M$_{w}$ by using empirical relations reported in literature (e.g., in the case of Central Italy seismicity we use the formulas contained in^55^, while the procedure suggested in^56^ is applied for New Zealand earthquakes and^57^ for events in Southern California).
- $\Delta$CFS stress and its uncertainty are calculated for each earthquake. Uncertainties of the stress values are estimated by propagation of the errors in the measure of spatial orientation of faulting and hypocentral parameters. The dominant contribution comes from the strike and dip angle errors, so that
  17 $$\begin{aligned} {\left\{ \begin{array}{ll} \varepsilon _{shear} \simeq \sqrt{\varepsilon _{+}^{2}\sin ^{2}\delta \cos ^{2}\delta + \varepsilon ^{2}_{\delta }\sigma _{+}^{2}\cos ^{2}\delta } \\ \varepsilon _{normal} \simeq \sqrt{\varepsilon _{+}^{2}\sin ^{4}\delta + \varepsilon ^{2}_{\delta }\sin ^{2}2\delta } \\ \varepsilon _{confinement} \simeq \frac{1}{3}\sqrt{\varepsilon _{+}^{2} + \varepsilon ^{2}_{-}}\approx 0.47\varepsilon _{+} \end{array}\right. } \end{aligned}$$
  where $\varepsilon$ represents the standard deviation to avoid misunderstanding with stress components $\sigma _{s}$, $\sigma _{c}$, $\sigma _{n}$ and $\sigma _{\alpha }^{(\pm )}$. $\varepsilon _{+}$ and $\varepsilon _{-}$ are the uncertainties of the positive and negative tangential stresses given by
  18 $$\begin{aligned} \varepsilon _{+} = \varepsilon _{-} \simeq \varepsilon _{\alpha }\sqrt{(2\sigma _{\theta \theta }\cos \theta \sin \theta )^{2} + (2\sigma _{\phi \phi }\cos \theta \sin \theta )^{2} + (2\sigma _{\theta \phi }\cos 2\theta )^{2}} \end{aligned}$$
  In the previous formulas, hypocentral uncertainties are not included for the sake of readability, since their contribution is subdominant. The epicentral uncertainty does not affect significantly the final output, while the depth error is more significant; however, both the aforementioned contributions are included.
- The correlation between the magnitude of the seismic events and the amplitude of the tidal stress is calculated over fixed time intervals $\Delta$t containing a minimum number of events (n$_{min} > 200$). This is to avoid large stochastic fluctuations that can otherwise occur when the sampling frequency is increased.
  19 $$\begin{aligned} \displaystyle \rho _{t_{n}} = \dfrac{\sum _{i = 1}^{N_{t_{n}}} \left( M_{wi}-\overline{M_{w}}\right) \left( \Delta CFS_{i}-\overline{\Delta CFS}\right) }{\sqrt{\sum _{i = 1}^{N_{t_{n}}} \left( M_{wi}-\overline{M_{w}}\right) ^{2} \sum _{k = 1}^{N_{t_{n}}}\left( \Delta {CFS_{k}}-{\overline{\Delta CFS}}\right) ^{2}}} \displaystyle \end{aligned}$$
  where $N_{t_{n}}$ is the number of failures occurred during the n$^{th}$ time step.

The associated uncertainty is simply obtained by propagation of magnitude and $\Delta$CFS errors.

### Duration of preparation time

We measure the duration T of preseismic trends in the correlation time series following the definition

20 $$\begin{aligned} T = t_{e} - t_{s} \end{aligned}$$

$t_{e}$ is the timing of the first large earthquake of the sequence, i.e., $M_{w}^{(mainshock)} - M_{w}^{(e)} \le$ 1.0 and $M_{w}^{(e)} \ge$ 4.5. Therefore, we do not measure the trend duration using the timing of the mainshock because foreshocks and preslip produce stress drop which drifts apart seismicity from the critical point, causing a drop in correlation (e.g., it is the case of L’Aquila and Amatrice-Visso-Norcia seismic sequence). $t_{s}$ is the timing in which the correlation trend begins, i.e., the lowest correlation value is observed. Time resolution cannot be so good because of the large number of earthquakes required for correlation estimation, so that the temporal uncertainty of $t_{s}$, $\sigma _{t} > 1$ year for single correlation time series. Therefore, the previous procedure for the estimation of $t_{s}$ is repeated by using several correlation time series generated changing the length of the time steps $\Delta$t, which is chosen so that each time interval contains a few hundred earthquakes ($\Delta$t $\sim$ 3-24 months, according to the number of events in catalog). The final value of $t_{s}$ is given by the average of the single estimations. This method guarantees $\sigma _{t} < 1$ year for high-quality seismic catalogs.

Once $t_{s}$ and $t_{e}$ are got, the preparation time and its uncertainty are obtained straightforwardly. In our analysis we also consider seismic events in which no preparatory period is highlighted as long as minimum requirements for the quality of the databases are guaranteed (e.g., 2000 South Iceland seismic sequence). In this case, T is evaluated assuming a uniform probability distribution for $t_{s}$ in the time interval in between the last two measures before the date of the mainshock, so that $T \sim \Delta$t/$\sqrt{12}$. Anyway, an increase in correlation $\rho$ is usually recorded at the time of the major event.

### b-value

The most common method for the estimation of b-value is based on the maximum likelihood technique^58,59^.

Unfortunately, the Aki−Utsu method is accurate only if $M_{w}^{max} - M_{w}^{min} \ge 3$^60^; moreover, it does not take into account the binning of magnitudes.

Since the first hypothesis is not always satisfied in our case, improved methods must be used. We apply the Tinti-Mulargia formula^61^, that is summarized by the following equations:

21 $$\begin{aligned} {\hat{b}}_{TM} = \frac{\ln \left( 1+ \frac{\delta _{M}}{\langle M_{w}\rangle - M_{wc}} \right) }{\ln (10) \delta _{M}} \end{aligned}$$

where $\delta _{M}$ is the binning interval for magnitudes, and

22 $$\begin{aligned} \sigma _{{\hat{b}}_{TM}} = \frac{\frac{\delta _{M}}{\langle M_{w}\rangle - M_{wc}}}{\ln (10) \delta _{M} \sqrt{N \left( 1+ \frac{\delta _{M}}{\langle M_{w}\rangle - M_{wc}} \right) }} \end{aligned}$$

In order to apply them, $\langle M_{w}\rangle$ and the completeness magnitude $M_{wc}$ must be calculated.

The first is straightforwardly got by definition of arithmetic average of recorded magnitudes, while the second is measured according to the Wiemer–Wyss method^62^.

## Conclusions

We develop a method in order to highlight different phases of the seismic cycle in fault systems by examining their response to well-known stress perturbations. We choose tides, whose effect on seismic modulation is investigated. Our analysis shows that the correlation between the amplitude of $\Delta$CFS and seismic energy rate usually increases before seismic sequences, while it progressively decreases during intense seismic activity. Swift drops are also observed while foreshocks and preslip occur. A preseismic phase, featured by increasing correlation, is detected before large and intermediate (M$_{w} \gtrsim 4.5$) shallow (depth $\le 50$ km) earthquakes in about $\sim$ 60$\%$ of cases, which is compatible with literature^29,30^. The duration of the anomaly is suggested to be related to the seismic moment M$_{0}$ of the impending mainshock through $T \propto M_{0}^{0.3}$ for M$_{0} \lesssim$ 10$^{19}$ N m, while the scaling exponent decreases for events of magnitudes above 6.5. The same power exponent, 1/3, is typical of seismic nucleation scaling of single seismic events^32^. This analogy could mean that the physical mechanism behind both these phenomena is unique. Consequently, the anomalies we measure might be interpreted as diffuse nucleation phases throughout the crust. Nevertheless, we analyze only a limited number of seismic sequences all over the world, so that further studies are required in order to corroborate our results. Moreover, the present technique is affected by large uncertainties, which do not allow to define risk levels and only fleeble hope exists that error bars can be significantly reduced in the future. Nonetheless, even though it cannot be of practical use for seismic hazard, our approach could illuminate slow hidden processes of progressive destabilization in the brittle crust. At last, the relation between frequency-magnitude scaling and stress perturbation is examined in depth. We show that the responsiveness of seismicity to stress modulations decreases as magnitude and depth increase, as suggested by numerical simulations^45^ and physical modeling^4^.
